# Role of obesity and hypertension in the incidence of atrial fibrillation, ischaemic heart disease and heart failure in patients with diabetes

**DOI:** 10.1186/s12933-021-01331-5

**Published:** 2021-08-04

**Authors:** Lucia La Sala, Antonio E. Pontiroli

**Affiliations:** 1 Lab. of Cardiovascular and Dysmetabolic Disease, IRCCS MultiMedica, Milan, Italy; 2 Dipartimento di Scienze della Salute, Università degli Studi di Milano, Milan, Italy

**Keywords:** Diabetes mellitus, Atrial fibrillation, Ischaemic heart disease, Heart failure, Hypertension, Obesity

## Abstract

In a cohort study performed using primary care databases in a General Practitioners Network, Groenewegen et al. report a clear association between diabetes and incidence of the major chronic progressive heart diseases, notably heart failure (Groenewegen et al. in Cardiovasc Diabetol 20:123, 2021). However, no mention is made of body mass index and hypertension in the methods or in the results. Obesity is linked to hypertension and hypertension is a major risk factor for all cardiovascular diseases, and prospective studies have shown that obesity and hypertension contribute significantly to atrial fibrillation in persons with diabetes. The data would be improved by assessing the role of obesity and of hypertension in the incidence of heart diseases in these patients. This would also lead to a better and personalized treatment of patients with diabetes, for instance through weight loss and intensification of treatment of hypertension, to modify the incidence of atrial fibrillation, ischaemic heart disease and heart failure.

Sir,

In a recent paper Groenewegen et al. report on the incidence of atrial fibrillation, ischaemic heart disease and heart failure in patients with diabetes [1] in the Netherlands. In a longitudinal cohort study performed using primary care databases in a General Practitioners Network, the authors show a clear association between diabetes and incidence of the major chronic progressive heart diseases, notably heart failure, with a more than twice increased risk. However, in this study no mention is made of body mass index in the “Methods” section or in the “Results” section.

Even though the primary interest was to describe the incidence of cardiovascular diseases in diabetes in the real world, the data would gain scientific value by dissecting the role of obesity itself in the incidence of cardiovascular diseases, possibly mediated by hypertension. In fact, obesity is associated with hypertension [2], and hypertension is a major risk factor for all cardiovascular diseases, including coronary heart disease, stroke, atrial fibrillation, heart failure, aortic and peripheral arterial disease, and valvular heart disease [3]. In the prospective cross‑sectional observational NOMED‑AF (Non‑ invasive Monitoring for Early Detection of Atrial Fibrillation) study performed in Poland, atrial fibrillation was observed in 25 % persons with diabetes as compared with 17% persons without diabetes [4]; also, persons with diabetes and atrial fibrillation had more frequently hypertension, obesity, and ischemic heart disease than persons without diabetes. There is no doubt that blood glucose levels represent a direct risk factor for cardiovascular diseases, of increasing value from prediabetes to diabetes, to insulin-treated diabetes [5, 6]. However, the cardiovascular risk gradually increases with increases in blood pressure regardless of the presence of and of the degree of glucose abnormality [7]. Finally, one should consider education and healthy habits of persons with diabetes; in Sweden, less educated people had a poor prognosis and a higher prevalence of CVD risk factors (including poor glycemic control, smoking and obesity) than well educated people [8]. Therefore, it seems wise to assume that diabetes is not a lonely actor responsible for atrial fibrillation, ischaemic heart disease and heart failure. The data of Groenewegen et al. [1] would be improved by assessing, likely through multivariable analysis, the role of obesity and of hypertension in the incidence of atrial fibrillation, ischaemic heart disease and heart failure in their patients. This would also lead to a better and personalized treatment of patients with diabetes, for instance through weight loss and intensification of treatment of hypertension, to modify the incidence of atrial fibrillation, ischaemic heart disease and heart failure [2].

## Data Availability

Not applicable.
